# Numerical simulations of the effects of regional topography on haze pollution in Beijing

**DOI:** 10.1038/s41598-018-23880-8

**Published:** 2018-04-03

**Authors:** Ziyin Zhang, Xiangde Xu, Lin Qiao, Daoyi Gong, Seong-Joong Kim, Yinjun Wang, Rui Mao

**Affiliations:** 1 Institute of Urban Meteorology, Chinese Meteorological Administration, Beijing, 100089 China; 2 Environmental Meteorology Forecast Center of Beijing-Tianjin-Hebei, Chinese Meteorological Administration, Beijing, 100089 China; 3 State Key Laboratory of Severe Weather, Chinese Academy of Meteorological Sciences, Beijing, 100081 China; 4 State Key Laboratory of Earth Surface Processes and Resource Ecology, Beijing Normal University, Beijing, 100875 China; 5 Korea Polar Research Institute, Incheon, 406-840 Korea

## Abstract

In addition to weather conditions and pollutant emissions, the degree to which topography influences the occurrence and development of haze pollution in downtown Beijing and the mechanisms that may be involved remain open questions. A series of atmospheric chemistry simulations are executed by using the online-coupled Weather Research and Forecasting with Chemistry (WRF-Chem) model for November-December 2015 with different hypothetical topographic height scenarios. The simulation results show that topography exerts an important influence on haze pollution in downtown Beijing, particularly the typical development of haze pollution. A possible mechanism that underlies the response of haze pollution to topography is that the mountains that surround Beijing tend to produce anomalous southerly winds, high relative humidity, low boundary layer heights, and sinking motion over most of Beijing. These conditions favor the formation and development of haze pollution in downtown Beijing. Furthermore, the reduction percentage in PM_2.5_ concentrations due to reduced terrain height in the southerly wind (S) mode is almost three times larger than that in the northerly wind (N) mode. In the context of the regional topography, the simple S and N modes represent useful indicators for haze prediction in Beijing to some extent, especially over medium to long time scales.

## Introduction

Beijing is located in the northern tip of the North China Plain and surrounded by mountains along the west, north and northeast. Beijing is the capital of China and one of the largest cities in the world; it has with approximately 20 million residents. Given the rapid urbanization and industrial development over the past several decades, Beijing and its adjacent areas are becoming more and more important in China and for the global economy. However, the rapid economic growth and urbanization have increased the level of air pollution in recent decades^1–8^. Beijing and eastern China have suffered from severe haze or smog days frequently in recent years. These events are characterized by high particle mass concentrations and low visibility. Severe haze pollution, exemplified by the persistent haze days in January 2013, represent a substantial threat to human health and traffic safety. These phenomena have stimulated great interest in studying the haze pollution in Beijing or eastern China as a whole^9–18^. Very serious haze pollution struck again in Beijing in November-December 2015, and heavy pollution red alerts were issued on December 7^th^ and 18^th^, 2015 (although, according to pollutant concentration records, these dates were not the worst cases over that period November-December 2015). That is the first time the capital issued a red alert (the most serious level) for air pollution in Chinese history^19–21^.

Haze pollution is generally attributed to the following two factors: the emission of pollutants to the lower atmosphere from fossil fuel combustion, construction and other sources and unfavorable meteorological conditions. Air quality and the occurrence of haze pollution are strongly influenced by meteorology. Meteorological factors have essential impacts on the accumulation or diffusion, spread and regional transport of air pollutants and have important impacts on the formation of secondary aerosols, which are generated by complex physical and chemical reactions^22–31^. In particular, weather conditions play an essential role in controlling the daily variability of air pollutant concentrations^5,32–34^. Previous studies have suggested that there is a close relationship between the occurrence of winter haze pollution in the Beijing-Tianjin-Hebei region and atmospheric circulation at middle to high latitudes over a long-term perspective^35–37^. In addition, on interannual time scales, the air pollution across central and eastern China or even South Asia in the winter and summer is closely related to the East Asian winter and summer monsoons, respectively^38–45^.

**Figure 1:**
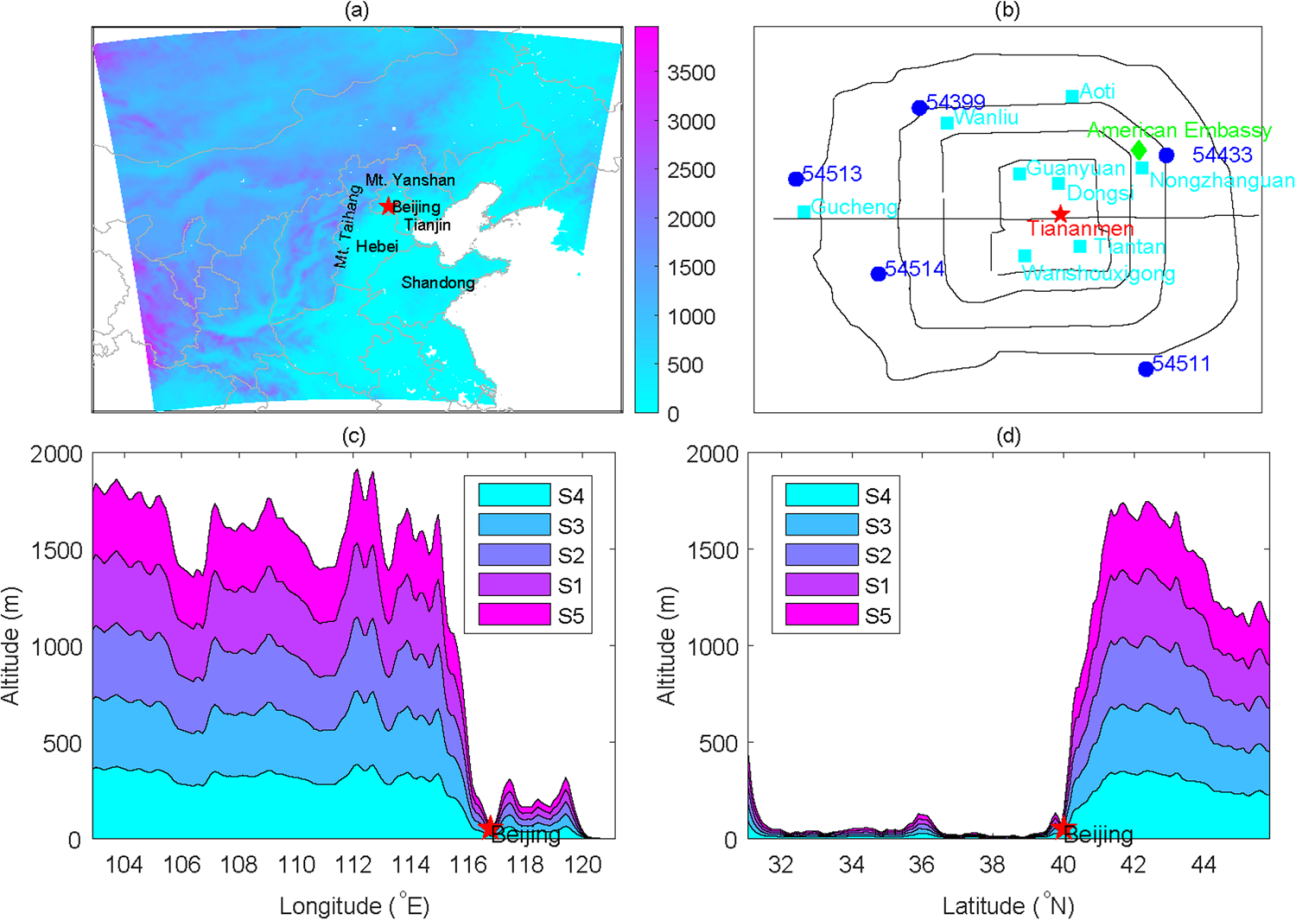
A map showing the topography of northern China and the spatial domain of the simulations (**a**); environmental monitoring sites operated by the Ministry of Environmental Protection of the People’s Republic of China (cyan squares) and American Embassy (green diamond) and meteorological stations operated by China Meteorological Administration (blue dots) in downtown Beijing (**b**); the topographic profiles of altitude-longitude along 39.5–40.5°N (**c**) and altitude-latitude along 116–117°E (**d**) in the different sensitivity simulations.

Terrain or topography may be another non-negligible factor that influences the haze pollution that occurs in Beijing and eastern China. For example, a previous study has suggested that the “harbor” effect on the westerlies in the eastern lee of the Tibetan Plateau’s large topography may be an important factor that influences the regional distribution of haze frequency in eastern China^46^. Beijing is located in the northern part of the North China Plain (Fig. 1a). The Taihang Mountains are located west of Beijing, whereas the Yanshan Mountains lie to the north and northeast of Beijing. Topographical profiles show that the altitude differences in both the west-east and north-south directions exceed 1000 m over very short horizontal distances (Fig. 1c,d). Thus, whether and how the regional topographic variations influence the occurrence and development of haze pollution in Beijing remain open questions. To date, few studies have examined the possible influence of topography on haze in Beijing or other areas quantitatively^47,48^. A close understanding of the effects of topography on air pollution assists in planning the spatial layout of industry areas and emission controls in Beijing and the adjacent areas and is also useful in improving the prediction skill achieved by weather and air quality forecasts produced by numerical models. This study aims to investigate the effects of topography on the occurrence and development of haze pollution using numerical sensitivity experiments.

## Results

### Analysis of observational data

**Figure 2:**
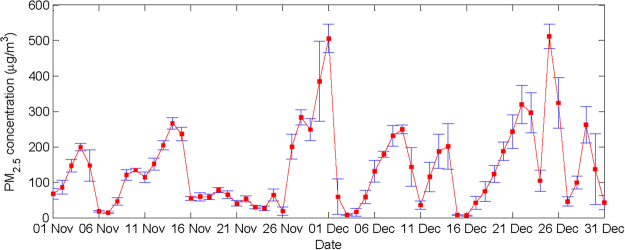
The daily mean PM_2.5_ concentrations in downtown Beijing over November-December 2015. Error bars denote the standard deviations calculated from 24 hours of recorded data.

The daily mean PM_2.5_ concentrations in downtown Beijing in November-December 2015 are presented in Fig. 2. The standard deviations determined from the 24-hour records from each day are indicated by error bars. Days with a high PM_2.5_ concentrations and a small error bars, such as December 1^st^ and December 25^th^, indicate heavy pollution throughout the day. In contrast, a large error bar generally indicates intense fluctuations in the PM_2.5_ concentrations, which may reflect either the accumulation or elimination of pollutants or both. It is clear that most (36 days, approximately 59%) of the daily PM_2.5_ concentrations exceed 75 μg/m^3^, which suggest polluted conditions, according to the ambient air quality standards of China. The mean concentration over this period was 140.6 μg/m^3^, which is far larger than the annual mean for 2015 (80.6 μg/m^3^, according to the Beijing Municipal Environmental Protection Bureau). During the November-December 2015, extreme severe pollution occurred, especially on the 1^st^ and 25^th^ of December, and the daily mean PM_2.5_ concentrations in downtown Beijing exceeded 500 μg/m^3^, which is very unusual. Generally, the high recorded PM_2.5_ concentrations indicate that severe or even extreme haze pollution occurred in Beijing in November-December of 2015.

### Modeling evaluation

**Figure 3:**
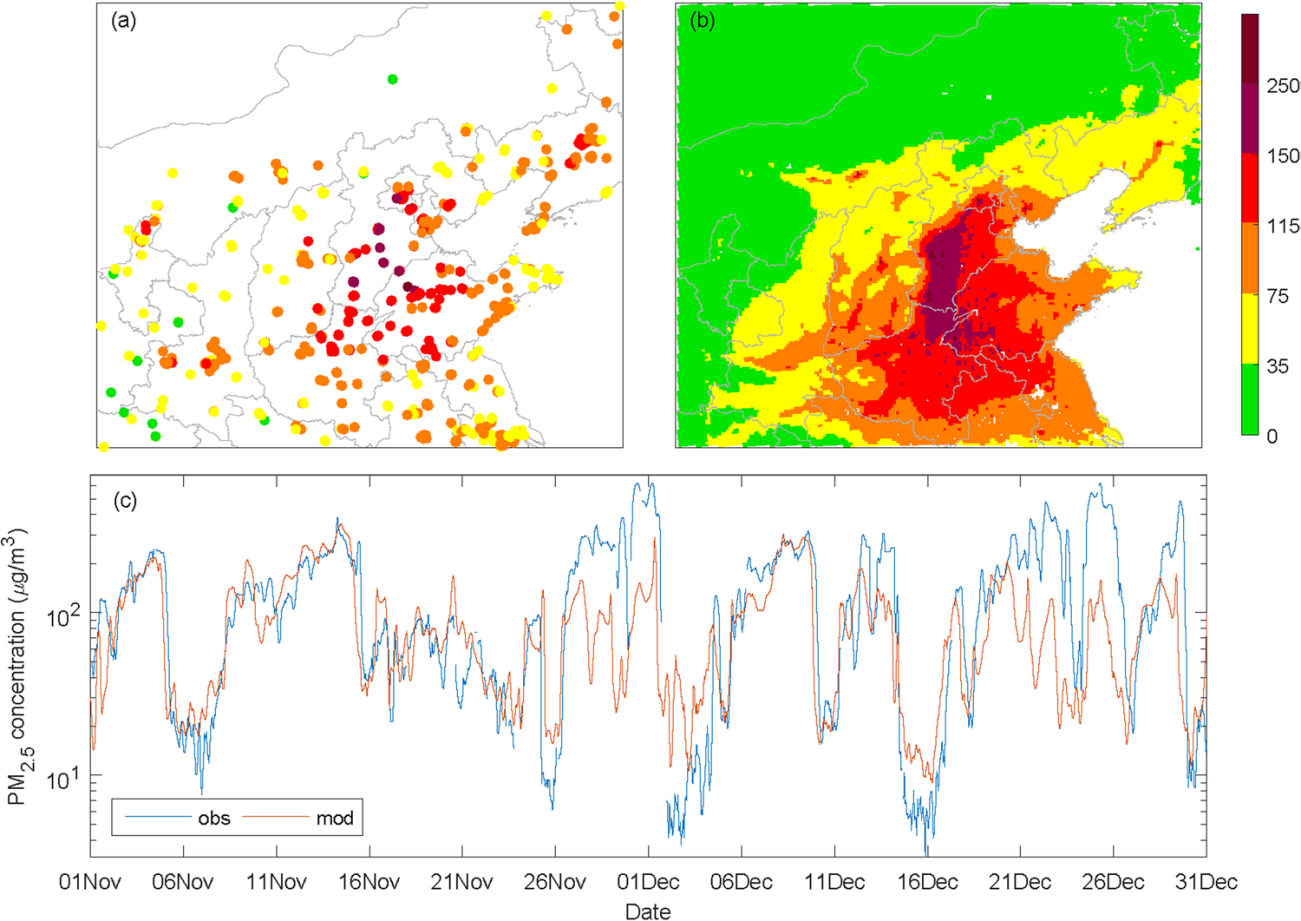
The spatial distribution of the observed (**a**) and simulated (**b**) mean PM_2.5_ concentrations during the period of November 1^st^ to December 31^st^ and the temporal variations in the observed and simulated hourly PM_2.5_ concentrations in downtown Beijing (**c**).

Using the Weather Research and Forecasting with Chemistry (WRF-Chem) model and output from the final (FNL) reanalysis data, a series of high-resolution simulation experiments is performed that focuses on the possible influences of topography on haze pollution in downtown Beijing during November-December 2015. We first compared the features of the spatial distribution of simulated PM_2.5_ concentrations to the observations during the study period. The observed and simulated mean PM_2.5_ concentrations over most of northern China during November-December 2015 are shown in Fig. 3a,b, respectively. As seen in the observations, most of Beijing, Tianjin, the south-central portion of Hebei province and the northwest part of Shandong province experience moderate (≥115 μg/m^3^) to high levels (≥150 μg/m^3^) of pollution during these two months. The spatial distribution of the simulated mean PM_2.5_ concentrations is generally consistent with that of the observations. Heavy pollution extends from the south-central portion of Hebei province along the foot of the Taihang Mountains to the Beijing areas. The spatial correlation coefficient between the observed mean PM_2.5_ concentrations at all of the stations (770 in total) located within the simulation domain and the corresponding PM_2.5_ concentrations derived from the simulations is 0.62 (p < 0.01). The temporal correlation coefficients between the observed and simulated PM_2.5_ concentrations are also calculated; on the daily and hourly time scales, the percentages of positive correlations are 93.8% and 94.2%, and the mean correlation coefficients are 0.34 and 0.29, respectively. These significant correlations suggest that the simulations generally capture the spatial distribution patterns and temporal fluctuations of the PM_2.5_ concentrations found in the observations over the entire areas. In downtown Beijing, the correlation coefficients between the observed and simulated PM_2.5_ concentrations are 0.48 and 0.46 on the daily and hourly time scales, respectively. Both of these correlations are significant at the 0.01 level. The comparison of the temporal variations in the observed and simulated hourly PM_2.5_ concentrations in downtown Beijing (Fig. 3c) indicates that the simulated PM_2.5_ variability is generally consistent with the observed, although the model does not capture the peak concentrations. Note that the skill of the simulation in representing the typical development of haze events (featured by gradual increases in PM_2.5_ concentrations) is better than its ability to represent the intense fluctuations in haze (featured by rapid increases or decreases in PM_2.5_ concentrations over very short time periods). Generally, the simulation results are reliable and useful in examining the effects of topography on the occurrence of haze pollution in Beijing.

### Sensitivity analysis

**Figure 4:**
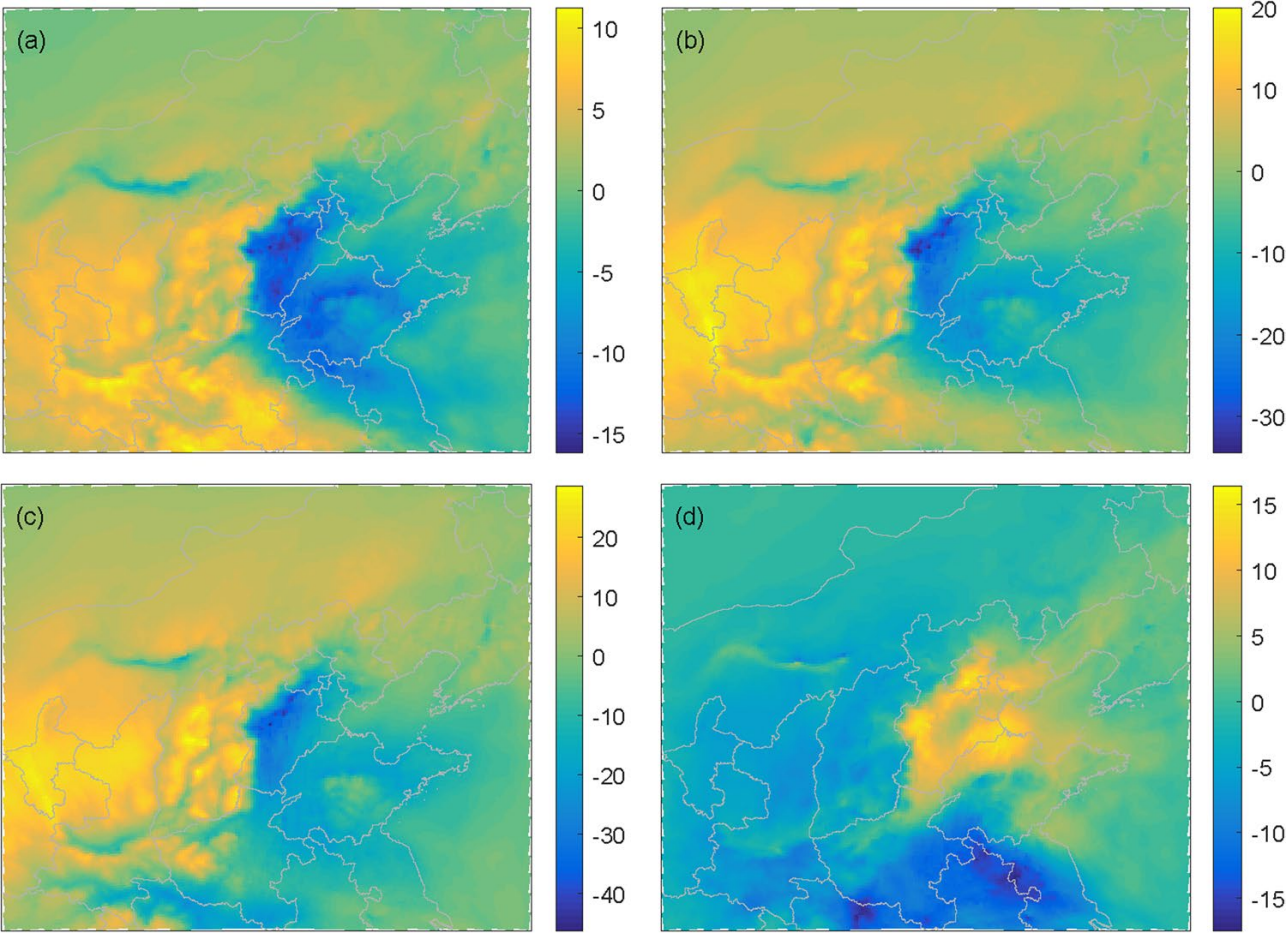
The spatial distribution of PM_2.5_ differences between scenarios S1 and S2 (S2-S1) (**a**), S3 (S3-S1) (**b**), S4 (S4-S1) (**c**), and S5 (S5-S1) (**d**) in μg/m^3^.

**Figure 5:**
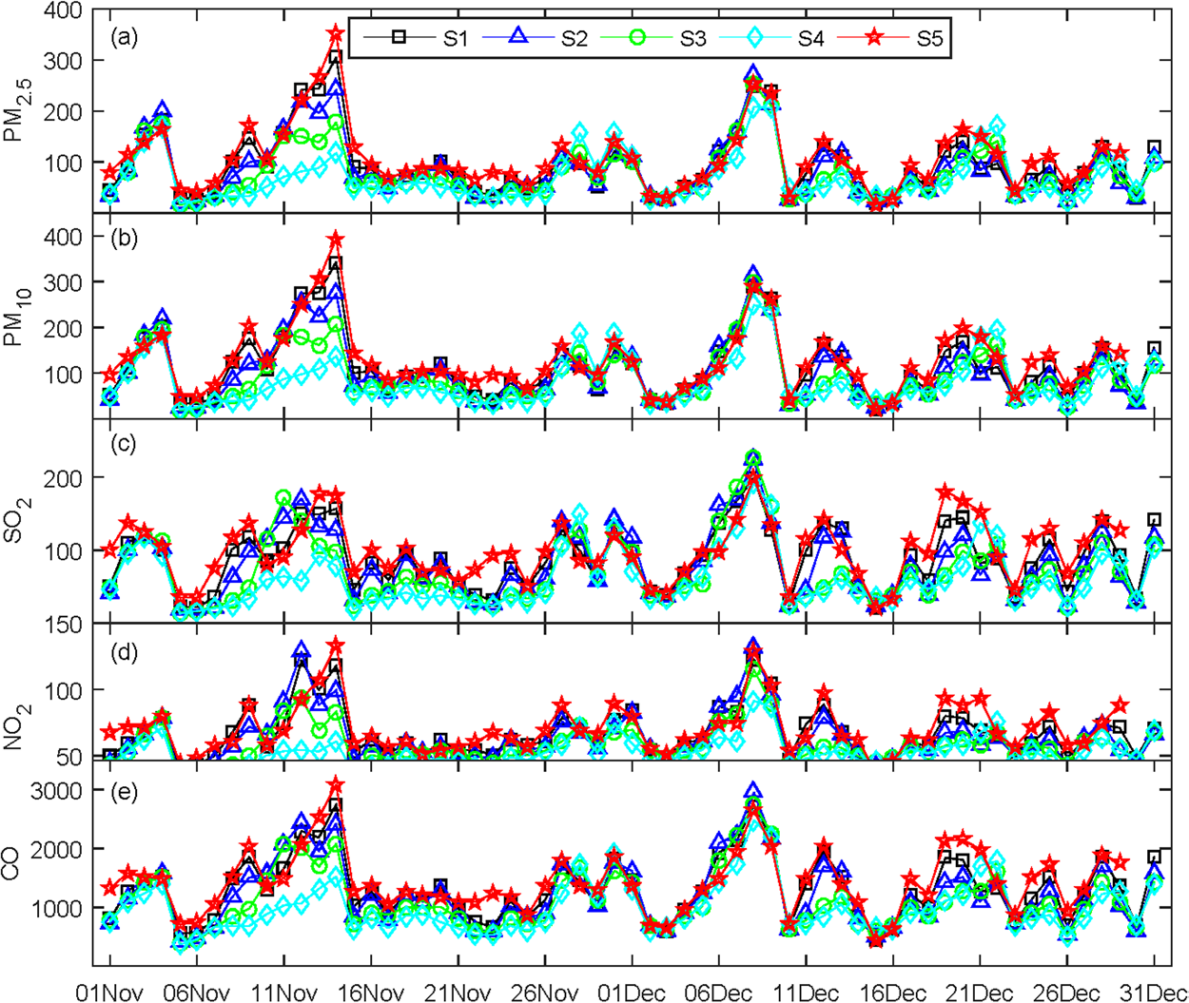
The daily concentrations of PM_2.5_ (**a**), PM_10_ (**b**), SO_2_ (**c**), NO_2_ (**d**) and CO (**e**) in downtown Beijing during November-December 2015 derived from the simulations using different scenarios in μg/m^3^.

We first examine the general characteristics of the response of PM_2.5_ concentrations to changes in topography. As the terrain heights drop, the PM_2.5_ concentrations decrease over most of the North China Plain, especially from south of Beijing to the south-central portion of Hebei province (Fig. 4a–c). In contrast, the PM_2.5_ concentrations clearly increase over most of the North China Plain as the terrain heights is increased (Fig. 4d). Moreover, the responses of other important pollutants, such as PM_10_, SO_2_, NO_2_, and CO, to topographic height changes are roughly consistent with those of PM_2.5_ (not shown). The daily variations in the concentrations of PM_2.5_, PM_10_, SO_2_, NO_2_, and CO in downtown Beijing from November to December 2015 derived from the simulations performed using different topographical scenarios are shown in Fig. 5. The pollutant concentrations generally increase as the terrain heights increases, especially during the typical development of haze, such as during the period of the 7^th^ to the 16^th^ of November (Fig. 5).

To quantitatively understand the influence of the regional topography on haze pollution in downtown Beijing in the different scenarios, the mean concentrations of PM_2.5_, PM_10_, SO_2_, NO_2_, and CO derived from all of the scenario simulations over the entire period and the typical haze events are calculated. When the topographic heights are scaled by 0.75 (S2), 0.50 (S3) and 0.25 (S4), all of the pollutant concentrations in downtown Beijing are clearly less than those in scenario S1 over both the entire period and the typical haze period. In contrast, when the height of the topography is scaled by 1.25 (S5), all of the pollutant concentrations in downtown Beijing are larger than these of S1 in both periods (Table 1). We further calculated the percentage changes in the pollutant concentrations in the scenarios with changed terrain heights (S2, S3, S4 and S5) relative to the scenario S1 (Table 2). During November to December 2015, the PM_2.5_ concentrations in downtown Beijing decrease by 9.2%, 18.3%, and 26.1% as the topographic heights are lowered by 25%, 50%, and 75%, respectively, whereas the concentration increases by 12.9% when the topographic height increases by 25%. During the typical development of haze, the PM_2.5_ concentrations appear to decrease by 15.3%, 36.5%, and 59.2% as the topographic heights decrease by 25%, 50%, and 75%, respectively; however, they increase by 10.4% when the topographic height increases by 25%. The percentage changes in the PM_10_ concentrations are very similar to the percentage changes in the concentrations of PM_2.5_, SO_2_, NO_2_, and CO. However, there is an exception; namely, no distinct changes in the NO_2_ concentrations occur in scenarios S5 relative to S1 during the period of November 7^th^ to 16^th^ (0.1%).

**Table 1:** Mean concentrations of the pollutants in the different scenarios.

Pollutant	Period	Scenario (μg/m^3^)				
		S1	S2	S3	S4	S5
PM_2.5_	Nov-Dec	94.9	86.2	77.5	70.1	107.2
	11.7–11.16	150.2	127.3	95.4	61.3	165.8
PM_10_	Nov-Dec	113.1	102.9	92.3	83.2	127.5
	11.7–11.16	173.2	148.7	112.9	73.1	191.2
SO_2_	Nov-Dec	87.9	81.4	71.7	62.0	99.8
	11.7–11.16	102.5	97.6	79.4	48.2	115.4
NO_2_	Nov-Dec	66.7	63.5	58.3	52.8	70.7
	11.7–11.16	79.1	75.1	61.9	46.7	79.2
CO	Nov-Dec	1308.8	1232.6	1120.4	1002.8	1431.4
	11.7–11.16	1664.6	1587.5	1342.3	930.9	1782.7

**Table 2:** Percentage changes in the pollutant concentrations among the different scenarios.

Pollutant	Period	Scenario (%)			
		(S2–S1)/S1	(S3–S1)/S1	(S4–S1)/S1	(S5–S1)/S1
PM_2.5_	Nov-Dec	−9.2	−18.3	−26.1	12.9
	11.7–11.16	−15.3	−36.5	−59.2	10.4
PM_10_	Nov-Dec	−9.0	−18.4	−26.4	12.7
	11.7–11.16	−14.2	−34.8	−57.8	10.4
SO_2_	Nov-Dec	−7.4	−18.4	−29.4	13.5
	11.7–11.16	−4.7	−22.5	−52.9	12.6
NO_2_	Nov-Dec	−4.8	−12.6	−20.7	6.0
	11.7–11.16	−5.0	−21.7	−40.9	0.1
CO	Nov-Dec	−5.8	−14.4	−23.4	9.4
	11.7–11.16	−4.6	−19.4	−44.1	7.1

In summary, the topography plays an important role in aggravating the concentrations of pollutants (especially the PM_25_ and PM_10_) in downtown Beijing during periods of air pollution, especially during the gradual development of haze pollution.

## Discussion

### Analysis of the relevant mechanisms

**Figure 6:**
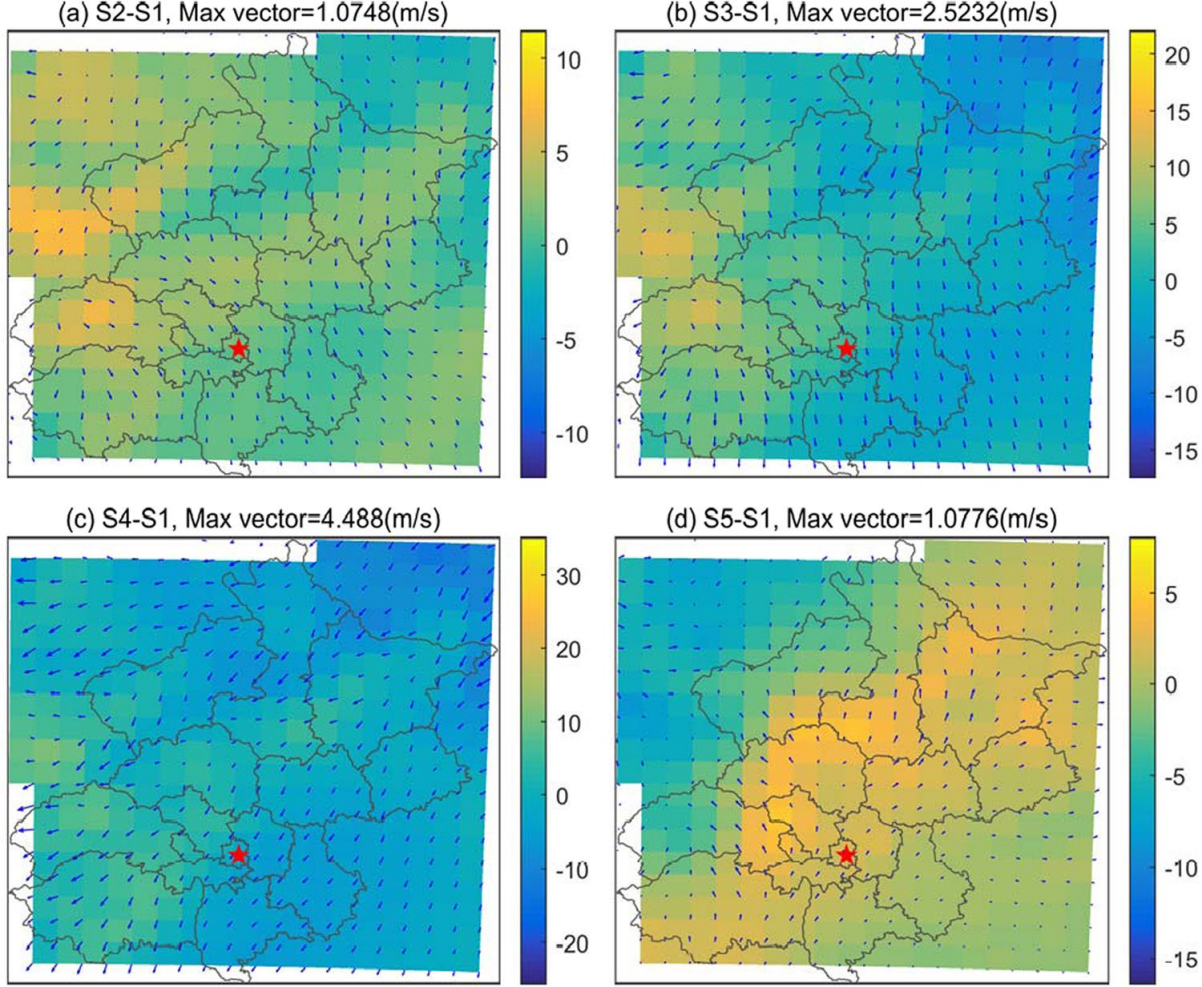
The composite anomaly fields of surface wind vectors and relative humidity for S2-S1 (**a**), S3-S1 (**b**), S4-S1 (**c**) and S5-S1 (**d**), respectively.

**Figure 7:**
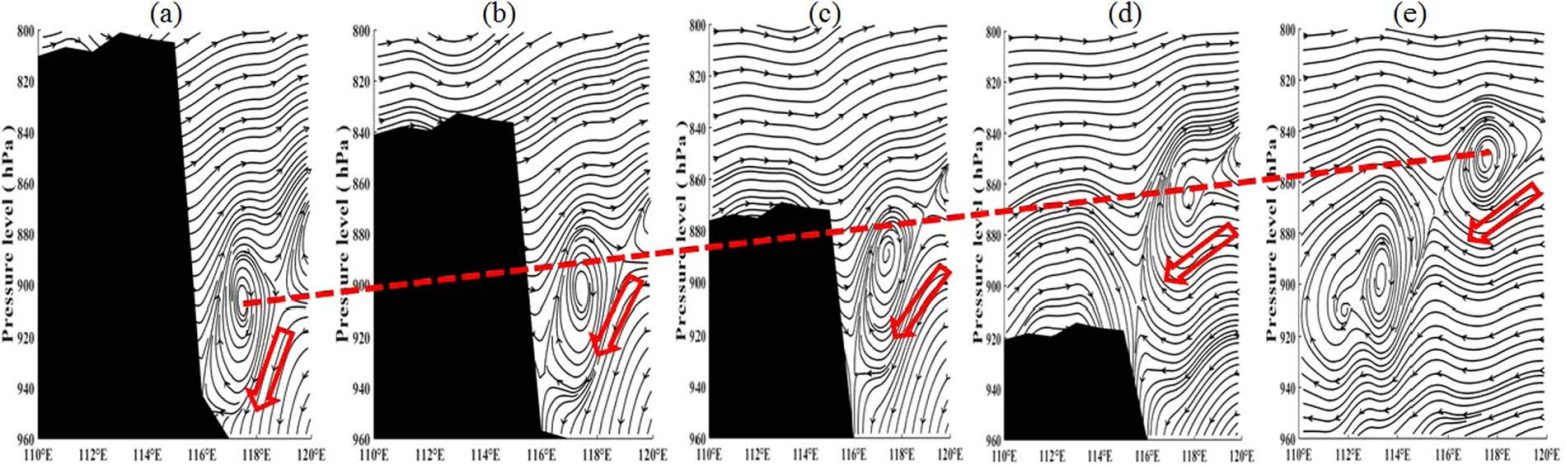
Profiles of simulated zonal and vertical winds along 39.5–40.5°N in the S5 (**a**), S1 (**b**), S2 (**c**), S3 (**d**), and S4 (**e**) scenario from November 7^th^ to 16^th^. The vertical winds have been multiplied by 400; the opaque regions denote the terrain.

The simulation results suggest that topography indeed exerts an important influence on air pollutant concentrations in downtown Beijing. We thus wonder what the possible mechanisms underlie the relationships and whether the modulation depends on the favorable weather conditions. First, we investigate the response of surface winds and relative humidity to the topographic changes. Fig. 6 shows the composite anomaly fields of surface wind vectors and relative humidity during November 7^th^ to 16^th^. The anomalous northerly winds and low relative humidity become dominant in Beijing and the surrounding areas as the terrain is lowered, especially when the terrain drops by 50% (Fig. 6b) and 75% (Fig. 6c). In contrast, most of the study area is dominated by anomalous southerly winds and high relative humidity when the terrain height increases (Fig. 6d). The anomalous southerly winds are likely accompanied by static winds, due to the blocking produced by the high topography to the north and west. A similar response is also found in the planetary boundary layer height (PBLH), which increases (decreases) over Beijing and its adjacent areas when the terrain height drops (increases). Furthermore, the profiles of zonal and vertical winds over central Beijing in the simulations that evaluate different scenarios are examined. The results show that increases in terrain height may lead to a sinking vertical vortex in the lower troposphere on the eastern lee of the regional topography, which promotes the development of sinking motion over downtown Beijing (Fig. 7). This sinking motion typically depresses the vertical exchange in pollutants and is often accompanied by or leads to a lower PBLH and weak winds. This phenomenon is similar to the “harbor” effect on the westerlies in the eastern lee of the Tibetan Plateau’s large topography, which may be an important factor that influences the regional distribution of haze frequency in eastern China^47^. Thus a simple conceptual model can be described as that increase in terrain height lead to anomalous southerly winds, high relative humidity, low boundary layer heights, and sinking motion, which favor the formation and development of haze pollution in Beijing, and vice versa.

### Implications of the S and N modes

**Figure 8:**
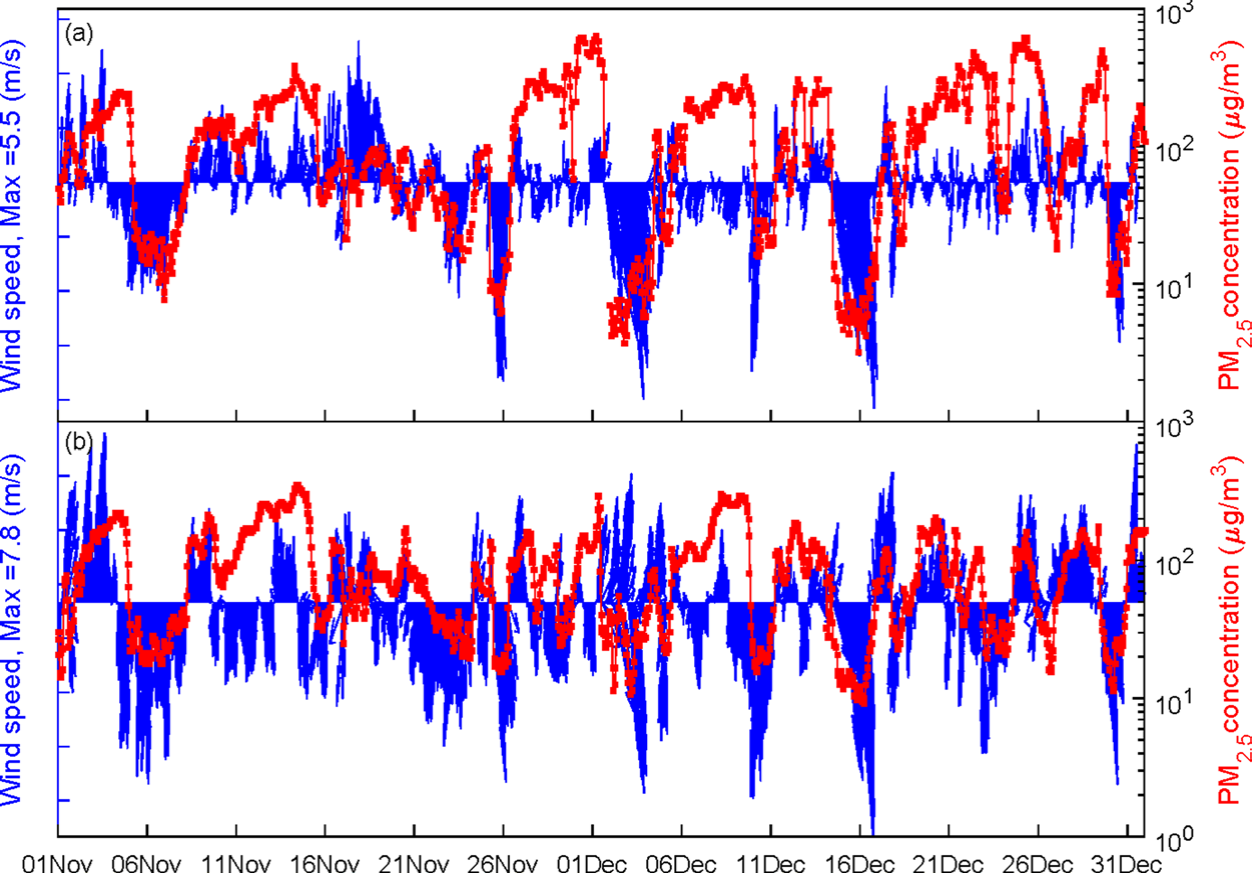
The observed (**a**) and simulated (**b**) PM_2.5_ concentrations and wind vectors in downtown Beijing.

The observed data show that the PM_2.5_ concentrations in downtown Beijing are distinctly related to the wind direction. The southerly and northerly winds correspond to high and low (or no) pollutions, respectively (Fig. 8a). The relationship between the winds and haze pollution in downtown Beijing is well reproduced in the atmospheric chemistry simulation (Fig. 8b). This result suggests that the simple southerly (S) and northerly (N) wind modes are very useful predictors of haze in downtown Beijing to some extent. Thus, it is of interest to understand the influence of topography on haze pollution in downtown Beijing under the different wind modes. The percentage changes in the pollutant concentrations in the different scenarios and the different wind modes are calculated (Table 3). For example, in the context of the N mode, the PM_2.5_ concentrations decreases by 5.0%, 9.6%, and 14.5% as the terrain height drops by 25%, 50%, 75%, respectively. However, in the context of the S mode, the PM_2.5_ concentrations decrease by 13.5%, 27.0%, and 37.6%, respectively. The reduction percentage in PM_2.5_ concentrations due to terrain height decreases in the S mode is almost three times larger than that in the N mode. This opposite relationship is noted when the terrain height increases by 25%. Similar characteristics are also found in the concentrations of other pollutants, such as PM_10_, SO_2_, NO_2_, and CO (Table 3). The response of pollutants concentrations to terrain height changes are closely related to the responses of meteorological variables, such as wind speed, relative humidity, and the PBLH. When the terrain height drops, the increases (decreases) in wind speed and the PBLH (RH) in the S mode are larger than those in the N mode, and vice versa (Table 4). Thus, characterizing the different modulating effects of topography on haze pollution in downtown Beijing under the S and N modes is important. This information would be very useful in producing medium-range to long-term (monthly to seasonal) predictions or evaluations of air pollution.

**Table 3:** Percentage changes (%) in the pollutant concentrations among the different scenarios and the different wind modes.

Pollutant	Wind mode	Scenario			
		(S2–S1)/S1	(S3–S1)/S1	(S4–S1)/S1	(S5–S1)/S1
PM_2.5_	S	−13.5	−27.0	−37.6	7.9
	N	−5.0	−9.6	−14.5	18.2
PM_10_	S	−13.4	−27.5	−37.8	7.5
	N	−4.4	−8.9	−14.6	18.4
SO_2_	S	−15.0	−31.5	−40.6	6.9
	N	1.8	−2.4	−15.8	22.1
NO_2_	S	−7.3	−17.6	−25.5	4.7
	N	−2.6	−8.2	−16.5	7.3
CO	S	−10.8	−23.6	−31.8	6.2
	N	−1.0	−5.4	−15.1	12.7

**Table 4:** Percentage changes (%) in the meteorological variables among the different scenarios and the different wind modes.

Pollutant	Wind mode	Scenario			
		(S2–S1)/S1	(S3–S1)/S1	(S4–S1)/S1	(S5–S1)/S1
Wind speed	S	19.8	44.0	49.6	−9.0
	N	12.8	35.2	39.3	−15.1
RH	S	2.2	7.9	9.6	−0.2
	N	7.1	12.6	14.5	−0.7
PBLH	S	1.6	9.0	42.6	−10.3
	N	1.4	3.3	8.7	−14.1

### Uncertainties

Moreover, note that some uncertainties exist in the qualitative response of haze pollution to terrain height changes, due to the different spatial scales involved. In principle, differences are expected between the influence of regional topography on haze pollution in downtown Beijing and the influence of national- or continental-scale topography. Thus, further study is needed to explore this issue.

## Conclusion

The possible effects of different topographic scenarios on haze pollution in downtown Beijing are quantitatively examined using the online-coupled WRF-Chem model, output from the FNL reanalysis obtained from the National Centers for Environmental Prediction (NCEP), high-resolution anthropogenic emissions data derived from the Multi-resolution Emission Inventory for China (MEIC) and the hourly PM_2.5_ records generated at surface stations. The results suggest that the terrain height indeed plays an important role in controlling the haze pollution in downtown Beijing, especially during the typical development of haze, which featured by a gradual increase in PM_2.5_ concentrations. Concretely, the PM_2.5_ concentrations in downtown Beijing decrease by approximately 9.2% (15.3%), 18.3% (36.5%), and 26.1% (59.2%) as the topographic height in most of northern China is lowered by 25%, 50% and 75%, and it increases by 12.9% (10.4%) as the topographic height is increased by 25% during November-December 2015 (during a typical haze event), respectively. The statistical relationships are consistent with the simple mechanisms demonstrated by these simulations. For example, increases in terrain height lead to anomalous southerly winds, high relative humidity, low boundary layer heights, and sinking motion in the lower troposphere, which favor to the formation and development of haze pollution in downtown Beijing, and vice versa. Moreover, the reduction percentage in PM_2.5_ concentrations due to reductions in terrain height in the southerly wind mode (S mode) is almost three times larger than that in the northerly wind mode (N mode), suggesting that the S and N modes represent good indicators of haze pollution in downtown Beijing in relevant operations or studies, especially over medium to long time scales.

## Methods

### Data and study area

The hourly concentrations of PM_2.5_ (particulate matter with aerodynamic diameters less than 2.5 μm) in downtown Beijing used in this study are derived from eight stations operated by the Ministry of Environmental Protection of the People’s Republic of China during November-December 2015 (Fig. 1b). Moreover, the PM_2.5_ concentration data measured at the American Embassy station are also used for comparison and to fill in missing data using regression. From November 1^st^ to December 31^st^ in 2015, the percentages of missing hourly PM_2.5_ concentration records at the American Embassy station and the average of the eight operational stations are 0.68% (10 hours missing) and 3.62% (53 hours missing), respectively. In fact, the hourly PM_2.5_ records from the American Embassy station are highly consistent with the average values for the eight stations. The correlation coefficient is 0.96, which is significant at the 0.01 level (i.e., p < 0.01). The difference in their mean values is only 0.81 μg/m^3^. To better represent the data across the city, we take the average records from the eight stations as the actual PM_2.5_ concentration for downtown Beijing in this study. The meteorological records from five stations in downtown Beijing are shown in Fig. 1b (solid blue circles). The spatial scope in Fig. 1a also represents the model domain of the WRF-Chem simulations. For the daily mean values calculated here, a meteorological day is defined as the 20:00–20:00 (local time) throughout the paper.

### Model and simulation design

To investigate the influence of topography on haze pollution in Beijing, a series of sensitivity simulations involving five scenarios of topographic changes are evaluated using version 3.7 of the online-coupled Weather Research and Forecasting with Chemistry (WRF-Chem) model^49–53^. The first simulation involves the real (unchanged) terrain heights (labeled as S1). In addition, the real terrain heights are then scaled by 0.75, 0.50, 0.25, and 1.25 in the second (S2), third (S3), fourth (S4), and fifth (S5) scenario simulations, respectively. The output from the final (FNL) reanalysis produced by the Global Forecast System (GFS) of the National Centers for Environmental Prediction (NCEP) is used as the lateral meteorological boundary conditions in the simulations. The temporal and spatial resolutions of the FNL data used here are 6 hours and 1.0° × 1.0°, respectively. The primary settings and schemes used in the WRF-Chem simulations are shown in Table 5. The schemes used in our simulations are similar to those applied in the previous studies^19,54,55^, with a few differences. To better capture the process of haze development near the surface, 13 levels are specified under 1,500 m. A monthly dataset that describes anthropogenic emissions in 2012 at high spatial resolution (0.1° × 0.1°) is used in the simulations and was provided by the Multi-resolution Emission Inventory for China (MEIC)^56,57^.

**Table 5:** List of the experimental settings and schemes used in WRF-Chem.

Simulation settings	Values
Domain size Horizontal resolution Vertical resolution Time step for physics Time step for chemistry	201 × 222 cells (> 3.2 million km^2^) 9 km 30 levels up to 50 hPa 45 seconds 3 minutes
**Physics option**	**Adopted scheme**
Microphysics Longwave radiation Shortwave radiation Surface layer Land surface Planetary boundary layer Cumulus parameterizations	WRF Single-Moment 6-class scheme Rapid radiative transfer model (RRTM) Goddard Monin-Obukhov similarity Noah Land Surface Model Yonsei University scheme (YSU) Grell 3D
**Chemistry option**	**Adopted scheme**
Photolysis Gas phase chemistry Aerosols Anthropogenic emissions Biogenic emissions	Fast-J photolysis Carbon Bond Mechanism, version Z (CBM-Z) Model for Simulating Aerosol Interactions and Chemistry (MOSAIC) with 4 sectional bins MEIC with a spatial resolution of 0.1°×0.1° Guenther (from the United States Geological Survey land-use classification)

All of the simulations cover November-December 2015 because a very serious haze pollution event struck Beijing in November-December 2015, and red alerts were issued for the first time in Chinese history, as mentioned in the Introduction. Both the typical development of haze events (which feature a gradual increase in PM_2.5_ concentrations, i.e., November 7^th^ to 16^th^) and the fast-changing haze events (which feature explosive growth and/or dramatic reductions in PM_2.5_ concentrations, i.e., November 29^th^ to December 2^nd^ and December 22^nd^ to 26^th^) occurred during this haze season. Thus, this study increase our understanding of the possible effects of topography on haze pollution in downtown Beijing under different weather and pollution conditions.
